# Marine Bioprospecting: Enzymes and Stress Proteins from the Sea Anemones *Anthopleura dowii* and *Lebrunia neglecta*

**DOI:** 10.3390/md22010012

**Published:** 2023-12-23

**Authors:** Santos Ramírez-Carreto, Beatriz Miranda-Zaragoza, Nuno Simões, Ricardo González-Muñoz, Claudia Rodríguez-Almazán

**Affiliations:** 1 Instituto Nacional de Salud Pública, Centro de Investigación Sobre Enfermedades Infecciosas, Av. Universidad #655, Santa María Ahuacatitlan, Cuernavaca C.P. 62100, Mexico; santos.ramirez@ibt.unam.mx; 2 Departamento de Micro y Nanotecnologías, Instituto de Ciencias Aplicadas y Tecnología, Universidad Nacional Autónoma de México, Cto. Exterior S/N, C.U., Coyoacán, Ciudad de México C.P. 04510, Mexico; beatriz.miranda@icat.unam.mx; 3 Unidad Multidisciplinaria de Docencia e Investigación en Sisal, Facultad de Ciencias, Universidad Nacional Autónoma de México, Puerto Abrigo s/n, Sisal C.P. 97356, Mexico; ns@ciencias.unam.mx; 4 International Chair for Coastal and Marine Studies, Harte Research Institute for Gulf of Mexico Studies, Texas A and M University-Corpus Christi, Corpus Christi, TX 78412, USA; 5 Laboratorio Nacional de Resiliencia Costera (LANRESC), Laboratorios Nacionales, CONACYT, Sisal C.P. 97356, Mexico; 6 Instituto de Investigaciones Marinas y Costeras, CONICET, Facultad de Ciencias Exactas y Naturales, Universidad Nacional de Mar del Plata, Dean Funes 3350, Mar del Plata C.P. 7600, Argentina; ricordea.gonzalez@gmail.com

**Keywords:** *Lebrunia neglecta*, *Anthopleura dowii*, proteome, crude venom extract, enzymes, sea anemone

## Abstract

The bioprospecting of sea anemone tissues and secretions has revealed that they are natural libraries of polypeptides with diverse biological activities that can be utilized to develop of biotechnological tools with potential medical and industrial applications. This study conducted a proteomic analysis of crude venom extracts from *Anthopleura dowii* Verrill, 1869, and *Lebrunia neglecta* Duchassaing & Michelotti, 1860. The obtained data allowed us to identify 201 polypeptides, of which 39% were present in both extracts. Among the obtained sequences, hydrolase-type enzymes, oxidoreductases, transferases, heat shock proteins, adhesion proteins, and protease inhibitors, among others, were identified. Interaction analysis and functional annotation indicated that these proteins are primarily involved in endoplasmic reticulum metabolic processes such as carbon metabolism and protein processing. In addition, several proteins related to oxidative stress were identified, including superoxide dismutase, peroxiredoxins, thioredoxin, and glutathione oxidase. Our results provide novel information on the polypeptide composition of the crude venom extract from sea anemones, which can be utilized to develop molecules for therapeutic tools and industrial applications.

## 1. Introduction

The ocean is an ecosystem that is rich in biodiversity, serving as a leading source of natural compounds that have been explored for decades. The marine environment offers excellent economic opportunities for the development of new natural products due to its biodiversity. Compounds of marine origin have become base scaffolds to generate new molecules with therapeutic potential for various diseases or industrial applications [1,2]. There is a continuous search for natural compounds because not all end up having an application. Continued marine bioprospecting efforts allow for the sustainable exploration of new species of marine organisms to generate alternatives to using marine resources [3,4]. There is a particular interest in species that live in extreme environments or undergo significant physical–chemical and biological changes that promote physiological modifications in the organisms [5,6,7]. Species inhabiting highly variable marine zones have adapted their molecular machinery to different and varying conditions of salinity, light, wave action, ultraviolet radiation, pressure, temperature, and biological and chemical interaction, leading to the production of metabolites with biotechnological potential [2,3,8,9,10].

Some marine invertebrates live in environments with significant physical and biological variations, generating natural products with new biochemical characteristics to adapt to these environments [2,3,5,11]. These organisms are fascinating for marine bioprospecting due to the potential for novel natural marine products to be found. Marine invertebrate populations that inhabit rocky coastlines increase their energy costs because they face variations in the type of substrate and an extensive diversity of physical and biological factors, such as exposure to waves, temperature, desiccation, salinity, oxygen, light, fixation surface, competition, and predation [12,13,14,15]. In addition, the interaction of the tides with the waves is part of a scenario of manifestations of various adaptations and a peculiar diversity of species. In contrast, protected habitats such as reefs are characterized by low hydrodynamic stress, sedimentation, and the stratification of coastal water, producing marked daily or seasonal changes in temperature, salinity, and nutrient concentrations; metabolic adjustments are minor [16,17,18]. However, climate change is a factor that adds to the stress faced by marine organisms, generating metabolic adjustments in response to temperature [19,20].

Numerous reports address the issue of the physiological changes that marine organisms can undergo with the physical, chemical, and biological changes that can occur in the ocean. When ocean temperature increases, the metabolic activity of invertebrates can increase two- to three-fold. In a parallel manner, stress due to environmental warming inhibits oxygen consumption and leads to significant changes in metabolic processes, stimulating CoxI (aerobic metabolism), HSP70 (stress response), and SOD (antioxidant molecule), and organisms such as the pelagic bigfin reef squid, *Sepioteuthis lessoniana*, respond rapidly to cellular and metabolic adjustments of these proteins [21]. Organisms that live in hydrothermal vents increase the production of superoxide dismutase and catalase [22]. The reduction in oxygen in water bodies globally due to climate change is a factor that generates hypoxic conditions, which reduces locomotion and metabolic rate and increases oxidative stress, affecting other physiological processes of organisms that must be adjusted to quickly adapt to those conditions [23]. Organisms such as crustaceans, which face changes in salinity, generate a stress response and, at the same time, metabolic disorders, affecting the production of chitin and the enzymes required to produce this [24,25].

These suggest that marine enzymes have evolved to adapt to diverse conditions, potentially resulting in better stability and specificity for substrates and presenting advantages over their counterparts. In recent decades, interest has been generated in studying polypeptides, including enzymes, from marine organisms with applications in various areas, such as in the treatment of diabetes [14], like anticoagulants, and in the treatment of metabolic disorders, among other clinical and industrial applications [26].

In recent decades, peptides and proteins from cnidarians, particularly sea anemones, have been shown to have important structural and functional characteristics that benefit human health [14,27,28]. Approximately 1200 sea anemone species are distributed across marine habitats worldwide [29,30]. Sea anemones are marine invertebrates living in various environments, such as hydrothermal vents, reefs, and intertidal zones. Sea anemones are animals with a simple anatomy and sedentary lifestyles, producing polypeptides with functions in fundamental processes like feeding, defense, protection, reproduction, symbiosis, and growth [29]. While sea anemones have long been recognized as rich sources of polypeptides with various pharmacological activities, it was not until a few years ago that proteomic and transcriptomic methods were implemented to explore the proteins in their secretions and tissues [29,31,32]. Bioprospecting in these venomous marine invertebrates has traditionally focused on polypeptides with neurotoxic [33,34] and cytotoxic properties [35], with little attention given to other components of biotechnological interest, such as enzymes, amylases [36], chitinases [37], proteases [29,31], oxidoreductases [38,39], heat shock proteins [40], and structural proteins [41], which have been identified in their crude venom extracts (CVEs) and secretions. These enzymes can potentially be used to develop tools for medical and industrial applications [42,43].

**Figure 1:**
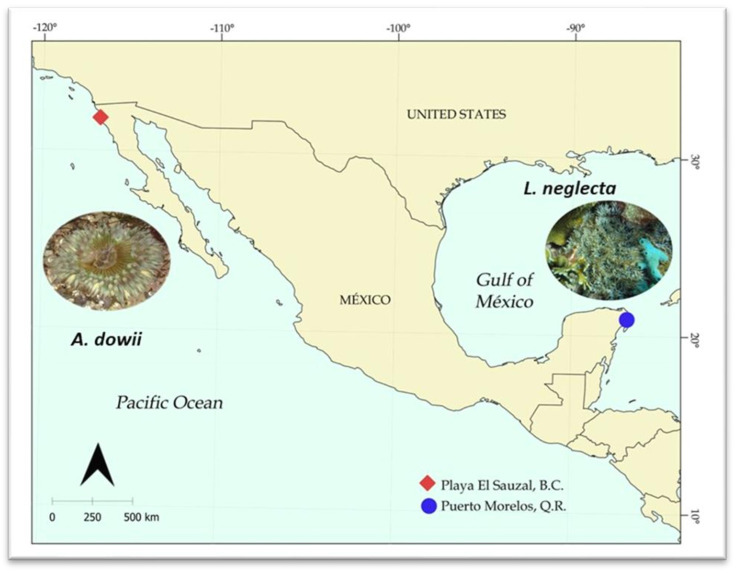
Sea anemone collection area. The red diamond indicates El Sauzal Beach, Baja California Norte, Mexico, where the *A. dowii* specimens were collected. The blue circle indicates the area where the Puerto Morelos reef is located, Quintana Roo, Mexico, where *L. neglecta* was collected.

In this study, marine bioprospecting was carried out involving proteomics on two sea anemones, *Anthopleura dowii,* located in the intertidal zone in Baja California Norte, Mexico, and *Lebrunia neglecta*, found in the reef area of Puerto Morelos, Quintana Roo, Mexico (Figure 1). The main goal of this study was to identify polypeptides (non-toxins) and provide new insights into engineering proteins for biotechnological applications with potential biomedical and industrial applications.

## 2. Results

The bioprospecting of *A. dowii* and *L. neglecta* sea anemones was carried out using proteomics of crude venom extracts, aiming to identify (non-toxic) proteins that the anemones produce and may be used in biotechnological applications.

### 2.1. Proteomic Analysis of Crude Venom Extracts (CVEs)

**Figure 2:**
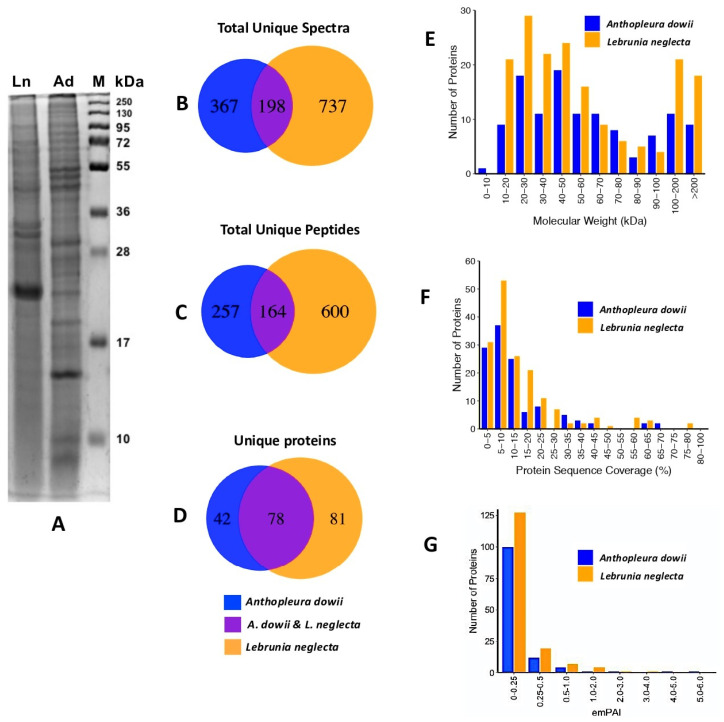
Proteomic comparison of crude venom extract from *Anthopleura dowii* and *Lebrunia neglecta*. (**A**) CVE electrophoretic profiles from *A. dowii* (Ad) and *L. neglecta* (Ln) samples analyzed with SDS–PAGE gel electrophoresis, 15% polyacrylamide, and Coomassie blue staining. (**B**–**D**) show Venn diagrams corresponding to the distribution of the number of unique spectra, unique peptides, and unique proteins, and the overlap between both samples. The distribution of the identified proteins with respect to their molecular weight, percentage of coverage, and the exponentially modified protein abundance index (emPAI) are shown in (**E**–**G**), respectively.

Specimens of *A. dowii* and *L. neglecta* were collected, and CVEs of both species were obtained, as indicated in Section 4.2. The electrophoretic profiles of the CVEs from *A. dowii* and *L. neglecta* exhibited differences in the region from 10 to 250 kDa (Figure 2A). These differences suggested the possibility of identifying disparities and similarities in the polypeptide composition between both CVEs when analyzed using Shotgun proteomics. Of the 8554 total spectra obtained through an MS/MS analysis of the three biological replicates for each CVE, 120 and 159 proteins were identified in *A. dowii* and *L. neglecta*, respectively (Supplementary Material S1).

In order to identify the proteins obtained in both CVEs, the Refseq_Cnidaria database was used, and the parameters are described in Section 4.5. Tryptic peptides obtained from the CVE from *A. dowii* were also aligned with the polypeptide sequences generated from the tentacle transcriptome [31,44], increasing the number of proteins identified in *A. dowii* and improving the subsequent annotation process. Among the two groups of proteins, we identified 201 unique proteins. Of these, 21% (42 proteins) were exclusively found in the *A. dowii* CVE, 40% (81 proteins) were exclusive to the *L. neglecta* CVE, and 39% (78 proteins) were found in both CVEs (Figure 2B–D). Using the Scaffold program, the proteomic indices of both species were obtained and compared to estimate differences and similarities. Our results show, in Venn diagrams, that the totals of unique spectra and peptides are more than double for the *L. neglecta* samples (Figure 2B,C), which coincides with the more significant number of unique proteins identified in *L. neglecta* compared to *A. dowii* (Figure 2D). The distribution of molecular weights, imPAI, and sequence coverage are similar between both samples (Figure 2E–G). Although the number of identified proteins is like that reported in other studies on sea anemones’ proteomics [31,45,46], we estimated that the identification rate was low, considering the complexity observed in the electrophoretic profiles of both CVEs. We speculate that the low identification and coverage rate could be due to the intrinsic characteristics of the sample that could complicate the identification process, as suggested by other research groups [45]. Therefore, we suggest that this type of sample may require a strategy that allows a larger amount of proteolytic peptides to be obtained for MS/MS sequencing analysis [47,48].

**Figure 3:**
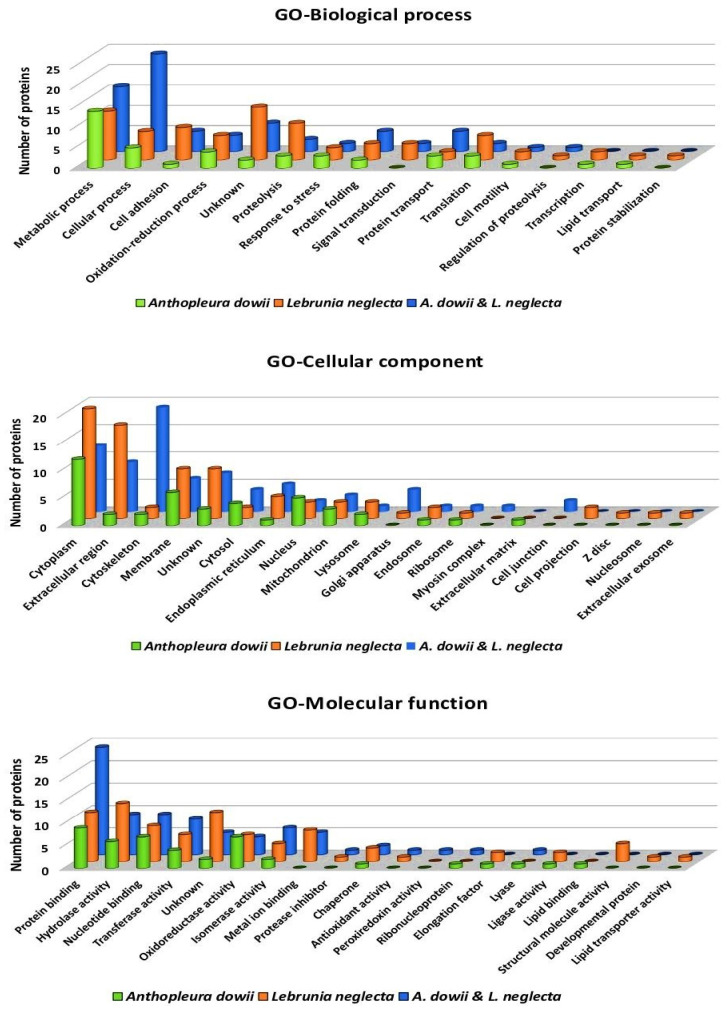
Annotation of the proteins identified in the crude venom extracts from *A. dowii* and *L. neglecta* by shotgun proteomics. They are classified into three main Gene Ontology (GO) categories (Supplementary Material S1): biological process, cellular component, and molecular function. The graphs show, on the *x*-axis, the sub-category of the identified proteins and, on the *y*-axis, the number of proteins identified for each sub-category. The annotation is based on amino acid sequence homology with respect to the proteins annotated in the UniProtKB database using BLASTP and QuickGO tools.

The identified proteins were annotated following the parameters and process indicated in Section 4.6. The information obtained from the automatic annotation using the Refseq_Cnidaria database was used and aligned with a homemade database generated from the *A. dowii* tentacle transcriptome, allowing, in some cases, for the complete sequence of the protein to be obtained and improving the annotation by submitting the sequence to the UniProt database. UniProt access codes were then subjected to GO analysis to classify proteins from both CVEs into the three main GO categories: “cellular components” (CC), “biological processes” (BP), and “molecular function” (MF) (Figure 3). The GO terms with the highest representation in the MF category were protein binding (GO:0005515), hydrolase activity (GO:0016787), and nucleotide binding (GO:0000166). In the case of BP, the most common GO terms shared between the samples from both CVEs were those related to metabolic processes (GO:0008152), cellular processes (GO:0009987), proteolysis (GO:0006508), and cell adhesion (GO:0007155). Our results predicted that most of the identified polypeptides are in the cytoplasm, extracellular region, and cytoskeleton (Figure 3). A Gene Ontology (GO) analysis of the *A. dowii* and *L. neglecta* proteomes revealed the presence of proteins previously reported in cnidarian secretions and found to be overrepresented under stress conditions, suggesting their role in protective mechanisms [40,49]. These proteins have also been documented in the sea anemone proteome of *Exaiptasia pallida* in the presence of the endosymbiont *Durusdinium trenchii* [50] and in the proteome of *Nematostella vectensis* under tissue damage conditions [40].

In order to identify the biological processes or metabolic pathways in which the protein components identified in both proteomes are participating, interaction maps were constructed for each group of proteins using the String v11.5 program, and the genome database of *N. vectensis* was used as a template [40]. Of the 119 and 158 proteins identified in *A. dowii* and *L. neglecta*, respectively, the amino acid sequences of 103 and 142 proteins were aligned with the *N. vectensis* database. Six and seven interaction groups were obtained in the proteomes of *A. dowii* and *L. neglecta*, respectively (Supplementary Material S2–S6). Group A in the *A. dowii* interactome comprises proteins involved in oxidation–reduction processes, such as mitochondrial superoxide dismutases (SODs), SODs of the Cu-Zn family, and peroxiredoxins-1. These proteins play a crucial role in cellular protection against the toxic free radicals generated within the cell [41]. Group B consists of proteins involved in protein processing and folding in the endoplasmic reticulum, including chaperones, thioredoxins, and heat shock proteins. Group C encompasses proteins related to metabolic processes such as glycolysis, gluconeogenesis, pyruvate metabolism, amino acid biosynthesis, and the pentose–phosphate pathway. Groups D and E include chitinase-type glycosidases and structural and adhesion proteins, respectively. Group F consists of proteins involved in signal transduction mediated by small GTPases. These proteins are predicted to participate in vesicular trafficking and are part of endocytosis, autophagy, and mitophagy.

In the case of *L. neglecta*, groups A and C comprise structural proteins and proteins related to cell adhesion. In Group B, we found that proteins participate in vesicular trafficking, while in Group D, the proteins are involved in the stress response, oxidation–reduction processes, and carbon metabolism. Group E has proteins that participate in translation, while group F consists of proteins related to fructose and mannose metabolism, the pentose phosphate pathway, and glycolysis/gluconeogenesis. Finally, group G includes proteins related to redox metabolism (Supplementary Material S6).

Although differences were observed between the number of unique proteins present in both CVEsamples (Figure 2D), the information generated from the annotation and the interactomes suggests that both samples are very homogeneous in terms of the presence of proteins with the same molecular function and their participation in biological processes. The most significant differences, attributed to the absence or presence of proteins with a specific function, are observed in those related to structural roles, lipid binding/transport, and development-related activities. Interestingly, some proteins identified only in *L. neglecta* were also found in the *A. dowii* tentacle transcriptome, suggesting that these proteins are present in *A. dowii* but were not identified in our proteomic analysis (see Supplementary Material S1).

### 2.2. Differentially Expressed Cellular Proteins

**Figure 4:**
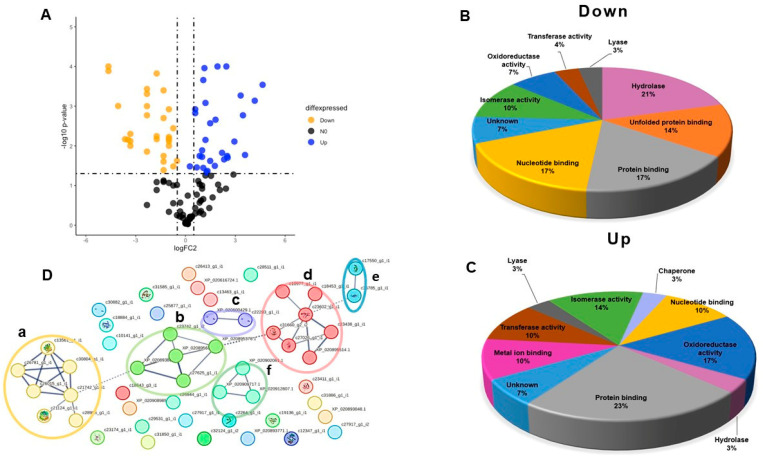
Differentially expressed proteins from proteomes of *A. dowii* and *L. neglecta*. (**A**) shows the volcano graph that represents the changes in the expression of 59 proteins present in both samples. The *x*-axis shows the log fold change of the *L. neglecta/A. dowii* ratio, while the *y*-axis shows the −log probability that was computed (*p*-value), associated with Student’s *t*-test. Orange dots indicate significantly downregulated proteins and blue dots indicate upregulated proteins. In (**B**,**C**), pie diagrams are shown with the lower and higher functional annotations, respectively, showing the differentially expressed proteins. In (**D**), protein–protein interaction network is shown. The circles indicate the interaction nodes and these are also indicated with parenthesis. a, proteins of sugar metabolism; b, proteins related to the stress response and the metabolism of reactive oxygen species; c, structural proteins; d, groups proteins that participate in protein folding; e and f, groups enzymes that participate in the modification of proteins and extracellular enzymes that act in the degradation of chitin, respectively.

The results of the identification and annotation of the proteomes provided sufficient information to determine the type of proteins that make up the CVEs of both anemone species. However, to estimate the differences between the proteomes related to the different ecological factors found in the environments where *A. dowii* and *L. neglecta* live, it was necessary to identify the differentially expressed proteins in both species. This information, in turn, allowed for the identification of metabolic pathways in which up- or downregulated proteins contribute to the knowledge of the physiological response of sea anemones to physical, chemical, and biological factors. When a double change-fold restriction was applied (see Section 4.5), 59 proteins showed significant changes in expression levels between both proteomes. A volcano plot was made considering up- and downregulated proteins (Figure 4), which allows us to quickly identify changes in large datasets consisting of experimental replicates [51]. In the volcano plot, each dot represents a protein. Proteins with positive and negative log2 (fold change) values, derived from the ratio (*Lebrunia neglecta/Anthopleura dowii*) between both samples, are averaged in triplicate; this corresponds to the value on the *x*-axis, and p-log10 values are indicated on the *y*-axis (proteins with high statistical significance). The cutoff line on the *x*-axis indicates a two-fold change in the intensity of the MS signals. This analysis led us to identify that, of the differentially expressed proteins, 29 were downregulated and 30 were upregulated.

The UniProt database and the Quick GO program classified these proteins based on their molecular function. The main molecular functional groups of proteins that significantly lowered their expression levels included catalytic proteins (45%), nucleotide binding (17%), protein binding (17%), unfolded protein binding (14%), and proteins with unknown function (7%) (Figure 3B). Catalytic proteins were also the leading upregulated group (47%), followed by protein binding (23%), nucleotide binding (10%), metal-ion binding (10%), function unknown (7%), and binding to unfolded proteins (3%) (Figure 4C).

In order to determine the types of protein–protein interactions among the 59 differentially expressed proteins, an interaction map was generated with String v11.5 software, as indicated in Section 4.6. Interaction networks showed thirty-seven interactions obtained from 51 altered proteins that matched the *N. vectensis* database using confidence mode (score > 0.7). We identified six interaction groups (Figure 4D). Group (A), indicated in yellow, comprises proteins related to sugar metabolism, and group (B), marked in lime green, comprises proteins related to the response to stress and metabolism of reactive oxygen species. Group (C), in purple, includes structural proteins with actin domains; group (D), indicated in red, includes endoplasmic reticulum proteins that participate in protein folding and cytoplasmic proteins that respond to cell stress. Groups (E) and (F), marked in blue and green, are enzymes that participate in the modification of proteins and extracellular enzymes that act in the degradation of chitin, respectively (see Supplementary Material S5). Our analysis shows an increase in the expression of oxidoreductase enzymes such as superoxide dismutase [Cu-Zn], peroxiredoxin-1 isoform X2, superoxide dismutase [mn], and endoplasmic reticulum proteins involved in protein folding and redox metabolisms, such as calreticulin and peptidyl–prolyl cis–trans isomerases, suggesting more significant activity of redox metabolism in *L. neglecta* compared to *A. dowii*. There is a decrease in the expression of proteins related to stress belonging to the heat shock protein 70 family in *L. neglecta* compared to *A. dowii*. A marked decrease in the expression of hydrolase-type enzymes was observed in the samples of *L. neglecta* compared to *A. dowii* (Figure 4B–D).

The results show the enrichment of proteins with enzymatic activities differentially regulated in both anemone species. This regulation could be related to the response of the anemone to exposure to environmental stressors that could be occurring in its habitats, such as temperature, ultraviolet radiation, changes in salinity, the availability of nutrients, and the accumulation of pollutants, among others.

### 2.3. Proteins with Many Biotechnology Applications

Some members of the protein families identified here have been isolated and functionally characterized from other organisms, including humans, and have shown potential as molecular tools for medical and industrial use. In order to gain more information about the observed proteins, a comparative analysis was carried out through the alignments of their amino acid sequences and homology models of each of them.

#### 2.3.1. Peptidases

**Figure 5:**
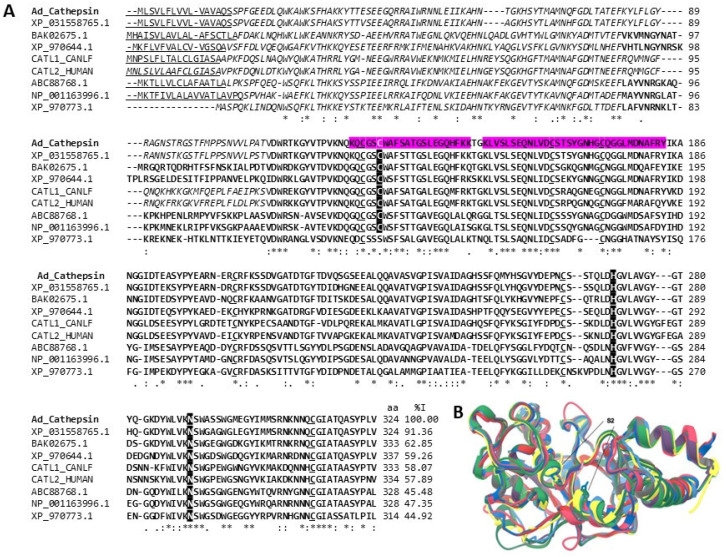
**Cathepsins-L**. (**A**) Alignment of amino acid sequences of putative Cathepsin-L from *A. dowii* (Ad_Cathepsin (c29945_g1_i1)) with Cathepsins-L from humans (Uni-ProtKB: CATL2_HUMAN), dogs (UniProtKB: CATL1_CANLF) and flour beetles (REF-SEQ: XP_0315589703_1, NP_001163996.1. GenBank: ABC88768.1, ABC88768.1, BAK02675.1). The region corresponding to the signal peptide is underlined, the propeptide is indicated in italics, and the region of the mature protein is indicated in bold. The residues that make up the active site are shaded black and highlighted with white letters. The cysteines that participate in the formation of disulfide bridges are underlined, and the region covered by the tryptic peptides in the Ad_Cathepsin sequence is highlighted by a magenta background. (**B**) Structure models of Cathepsin. Ad_Cathepsin is blue, XP_031558765.1 is yellow, BAK02675.1 is navy blue, XP_970644.1 is green, CATL1-CANLF is medium gray, CATL2_HUMAN is purple, ABC88768.1 is light gray, NP_001163996.1 is red, and XP_970773.1 is pink. The comparison of the Cα chains presented RMSD values that oscillate between 0.483 and 0.558.

Peptidases have proven helpful as additives in detergents, in the generation of products implemented in the pharmaceutical and cosmetic industries, in analytical processes such as treating samples for proteomic analysis, and in the food industry [42,43,44]. In the CVE of *A. dowii,* we identified a probable cysteine protease with 16% coverage in its sequence. The tryptic peptides matched the precursor sequence of a likely cysteine protease from our tentacle transcriptome database, and we named it Ad_Cathepsin (Id code: c29945_g1_i1. Supplementary S1). The precursor sequence showed 91.3% identity with a probable Cathepsin from the sea anemone *Actinia tenebrosa*, 58.3% with Cathepsin L2 from humans, and 58.2% identity with Cathepsin L from dogs (Figure 5A). In addition, it shares between 62.8 and 44.9% identity with proteases isolated from flour beetle genera, *Tribolium* and *Tenebrio*, which have been used in the development of formulations for the degradation of gluten present in some foods such as cereals [52,53]. Gluten triggers dermatitis herpetiformis and celiac disease in people intolerant to gluten. Because gluten is a common protein in foods, it is difficult for intolerant people to consume it, and they often suffer relapses due to its inadvertent intake [52,53]. The pre-treatment of food with proteases has proven not to be sufficient, which is why formulations that can be administered orally to patients are sought. However, exogenously administered proteases must possess a wide range of stability and activity to be useful at a gastric pH, show resistance to degradation by gastrointestinal tract proteases, and have favorable kinetics to allow for gluten degradation before gastric emptying [54]. Therefore, searching for new proteases that can function under certain environmental conditions is still necessary. The structural alignment presented in Figure 5B shows that Ad_Cathepsin maintains folding, made up of the characteristic domains of the papain superfamily [52]. The structural alignment shows that the most significant differences between these proteins are found in the region that joins the domain; on the contrary, the regions that bind to the substrate, S2 and P2, are highly conserved [55] and maintain residual Cys25 and His163 (Figure 5A,B).

#### 2.3.2. Chitinases

Chitinases catalyze the cleavage of β-1,4-O-glycosidic bonds in chitin, a polysaccharide that is abundant in nature and present in various pathogenic organisms for man, such as fungi, protozoa, and helminths [56,57,58]. Chitinases have anti-inflammatory properties [59] and can help heal wounds [57]. Since the cuticle of arbovirus vector mosquitoes is made of chitin, chitinases can also be used as bioinsecticides for the biological control of *Aedes aegypti* mosquitoes [56]. In addition, they can be used to control phytopathogens, and transgenic plants and recombinant strains of *Bacillus* can be generated that allow for the control of insects and fungi [57].

**Figure 6:**
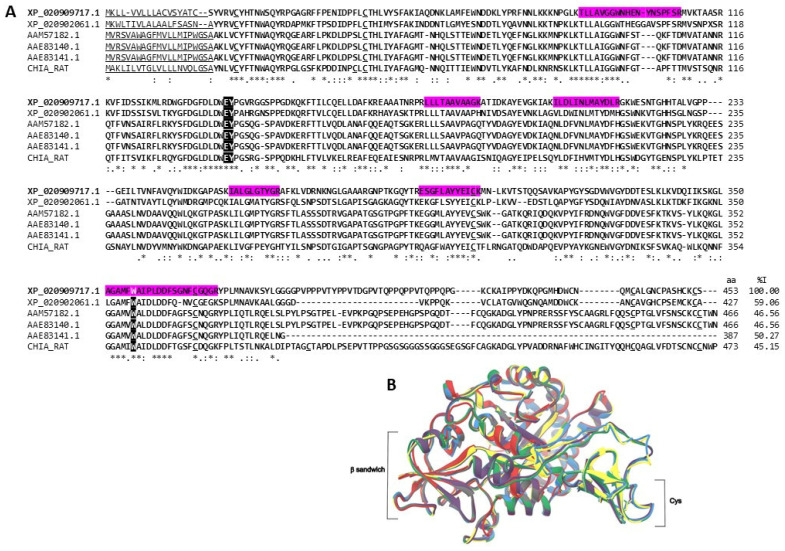
**Chitinases**. (**A**) Alignment of the amino acid sequences of the putative *Exaiptasia pallida* Chitotriosidase-1-like (XP_020909717.1) with chitinases from rats (Uni-ProtKB: CHIA_RAT), sea anemones (REFSEQ: XP_020902061.1) and humans (GenBank: AAM57182.1, AAE83140.1 y AAE83141.1). The magenta background shows the region covered by the tryptic peptides obtained from the proteomes of *A. dowii* and *L. neglecta*. The region corresponding to the signal peptide is underlined and the region of the mature protein is indicated in bold. The residues that make up the active and chitooligosaccharide binding sites are shaded black and highlighted with white letters. Cysteines involved in disulfide bond formation are underlined. (**B**) Structure models of chitinase: XP_020909717.1 is shown in green, XP_02092061.1 is in red, AAM57182.1 is yellow, AAE83140.1 is represented in gray, AAE83141.1 is blue, and CHIA_RAT is in purple. The comparison of the Cα chains presented RMSD values that oscillate between 0.127 and 0.616.

The oligosaccharides generated from the degradation of chitin, such as N-acetylglucosamine, chitooligosaccharides, glucosamine, and chitosan, may have applications in the pharmaceutical and food industries [60]. Chitooligosaccharides have even been reported to have antitumor activity [57]. In the extracts of both sea anemone species, six probable chitinases were identified (Supplementary Material S1). The chitinase identified in the *A. dowii* tentacle transcriptome does not correspond to the amino acid sequences identified in the proteome of the *A. dowii* tentacle transcriptome; this suggests that the chitinases identified here are produced in anatomical regions other than the tentacles [38]. With 32% coverage, a sequence like Chitotriosidase-1-like from *Exaiptasia pallida* (XP_020909717.1) was identified (Figure 6A), a good candidate for intervention in infectious diseases caused by chitin-containing pathogens [61]. Chitotriosidases comprise a catalytic domain and a chitin-binding domain, with distorted folding of the b-sandwich [62]. Additionally, these proteins retain six catalytic cysteines, which are essential for maintaining conformation and disulfide bonds [62]. The structural overlap of the homology model of Chitotriosidase-1-like from *Exaiptasia pallida* (XP_020909717.1) presented RMSD values between 0.127 and 0.616 (Figure 6B).

#### 2.3.3. Antioxidant Enzymes

Reactive oxygen species (ROS), such as hydrogen peroxide, superoxide anions, and hydroxyl radicals, are generated under physiological conditions because of cellular metabolism [61]. Although ROS play an essential role in cellular processes such as signal transduction, gene expression, enzyme regulation, and immune responses, when these oxygen species increase beyond equilibrium, cells undergo oxidative stress, leading to damage to proteins, lipids, and nucleic acids (DNA and RNA) [62]. This cell damage is related to various pathologies, such as cancer, diabetes, Alzheimer’s disease, Parkinson’s disease, and Amyotrophic Lateral Sclerosis [62,63]. To conserve balanced ROS levels, organisms are endowed with various enzymes that function as antioxidants in cells; among these are peroxiredoxins, thioredoxins, catalases, and peroxide dismutases [63,64]. Sequences encoding probable antioxidant enzymes, such as those mentioned above, were identified in the proteomes of *A. dowii* and *L. neglecta* (Supplementary Material S1).

Peroxiredoxins (Prxs 1-6) can catalyze the reduction of H_2_O_2_, peroxynitrite, and alkyl hydroperoxides [61,65]. Their active sites contain a Cys (Cys peroxidative, CP-SH) and function as a site of oxidation via peroxides generating sulfenic acid (CP-S-OH), which reacts with another Cys residue, called cysteine resolution (CR), to form a water molecule and a disulfide bond that can be further reduced through an electron donor, completing a catalytic cycle [61]. There are two groups of Prxs in mammals, 2-Cys and 1-Cys; this classification is according to the requirement for the Cys residue to complete the catalytic cycle [65]. However, Prx6 is the only one that does not have a resolution Cys and uses glutathione (GSH) to complete its catalytic peroxidative reaction. Therefore, it is a Prx 1-Cys. Furthermore, Prx6 is a moonlighting enzyme as it also possesses calcium-independent phospholipase A2 and lysophosphatidylcholine acyltransferase activity [66].

**Figure 7:**
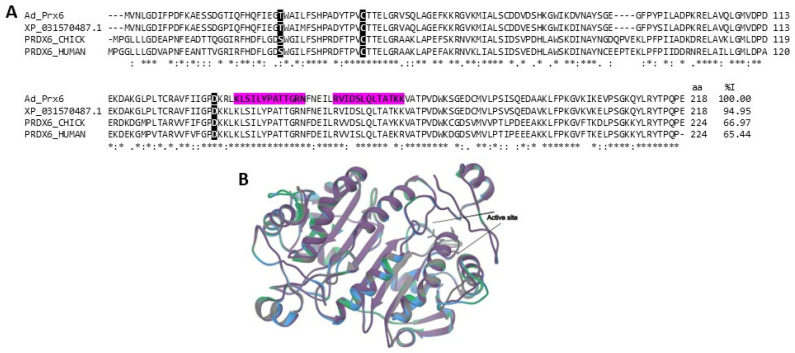
Peroxiredoxin-6 (Prx-6) alignment. (**A**) Alignment of the amino acid sequences of the putative Prx-6 from *A. dowii* (Ad_Prx6 (Transcriptome: c26896_g1_i1)) with Prx-6 from chickens (UniProtKB: PRDX6_CHICK), humans (UniProtKB: PRDX6_HUMAN), and from the sea anemone *Actinia tenebrosa* (REFSEQ: XP_031570487.1). The region covered by the tryptic peptides obtained in the *A. dowii* proteome is presented on a magenta background. On a black background with white letters, the residues related to Phospholipase A2 activity and peroxidative cysteine are highlighted. (**B**) Structure models of PRDX: Ad_Prx6 is gray, XP_031570487.1 is purple, PRDX6_CHICK is green, and PRDX6_HUMAN is blue. The comparison of the Cα chains presented RMSD values that oscillate between 0.032 and 0.342.

A probable Prx6 with sequence similarity to human and chicken Prx6 was identified in the *A. dowii* proteome and transcriptome and was named Ad_Prx6 (Figure 7). The precursor sequence of Dw_Prx6 also showed about 95% identity with *Actinia tenebrosa* peroxiredoxin-6-like (Figure 7A). Analyzing peroxiredoxin models, we observe that Dw_Prx6 contains a thioredoxin fold conserving the active site and the conserved Thr-Pro-Val-Cys-Thr-Thr motif (Figure 7B) [67]. An experimental evaluation of exogenous Prx6 as a radioprotective agent was carried out in mice, reducing damage by ionizing radiation, generating an increase in the survival rate of animals exposed to lethal and sub-lethal doses [68,69], and establishing the possibility of being able to design Prx-based preparations to protect patients undergoing radiotherapy.

Another type of enzyme with antioxidant properties is superoxide dismutase (SOD). These proteins are metalloenzymes that catalyze the conversion of the free radical superoxide anion (•O^2−^) into hydrogen peroxide (H_2_O_2_) and molecular oxygen (O_2_). Depending on the cofactor metal present in the active site, they are classified into four groups: Copper–Zinc-SOD (Cu, Zn-SOD), Iron SOD (Fe-SOD), Manganese SOD (Mn-SOD), and Nickel SOD. The therapeutic potential of SOD as an anti-inflammatory and anticancer agent has been suggested [70,71]. We identified a Copper–Zinc-SOD and a Mn-SOD in the *A. dowii* and *L. neglecta* proteomes. Mn-SOD was also identified in the *A. dowii* transcriptome and was named Ad_SOD [Mn].

**Figure 8:**
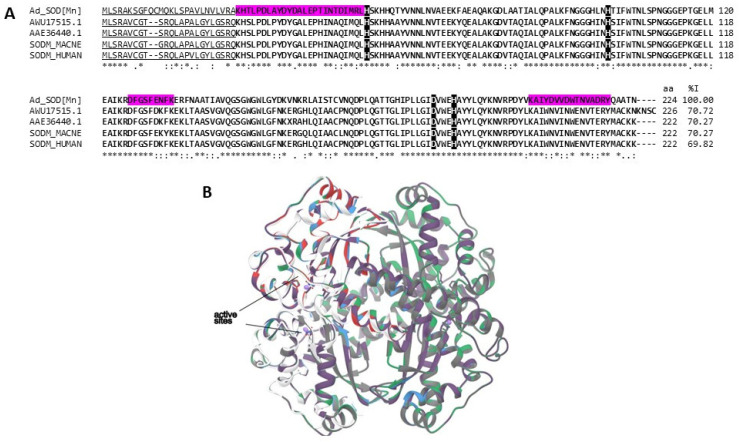
Superoxide dismutase [Mn] alignment. (**A**) Multiple alignment of the amino acid sequence of the putative mitochondrial superoxide dismutase from *A. dowii* (Ad_SOD [Mn] (Transcriptome: c27625_g1_i1)) with other human SOD2s (UniProtKB: SODM_HUMAN, GenBank: AWU17515.1 and AAE36440.1) and from pig-tailed macaques (UniProtKB: SODM_MACNE). The region covered by the tryptic peptides obtained from the proteomes of *A. dowii* and *L. neglecta* is presented on a magenta background. On a black background with white letters, the residues related to the binding to manganese are highlighted. The region corresponding to the transit peptide is underlined and the region of the mature protein is indicated in bold. (**B**) Structural models of SOD. Ad_SOD [Mn] is red, AWU17515.1 is blue, AAE36440.1 is gray, SODM_MACNE 551 is purple, and SODM_HUMAN is green. The comparison of the Cα chains presented RMSD values that oscillate between 0.014 and 0.536.

Ad_SOD [Mn] presented between 70.2 and 69.8% identity in its amino acid sequence with the SOD2 sequences of humans (UniProtKB/Swiss-Prot: P04179.1) and the macaque *Macaca nemestrina*, respectively (Figure 8A). Furthermore, it showed 70.7 and 70.2% identity with AWU17515.1 and AAE36440.1. The structural analysis showed that the SODs displayed manganese SOD folding [72]. SODs present the highest structural homology between each other, showing the lowest RMSD values (0.014–0.536), conserving the structure and geometry of the active site (Figure 8B). The latter is human SOD2, differing from SOD2 P04179.1, and their recombinant forms can internalize leukemic T cells, generating apoptosis in 99% of them without causing toxic effects in healthy cells [70]. SOD enzymes have shown good potential for use in the design of therapeutic molecules for the treatment of cancer and other pathologies related to oxidative stress; however, exogenous SOD has shown low bioavailability, mainly when applied orally, since the acidic pH and the proteases of the stomach cause its denaturation and degradation [71]. That is why the bioprospecting of SOD enzymes from different natural sources is essential to obtain possible candidates that are more stable at certain pH conditions and less vulnerable to proteolysis. This type of enzyme is used for veterinary use and in food supplements [71].

#### 2.3.4. Heat Shock Proteins (HSPs)

Heat shock proteins (HSPs) are highly conserved proteins that are inducible by a variety of stressful stimuli and by physiological processes, including cell differentiation and development [70]. At the cellular level, Hsp70 proteins are essential for proteostasis, aiding in the folding of newly synthesized and denatured proteins and, if necessary, in the degradation of aggregated proteins [73]. HSPs are classified into different classes based on their molecular weight, and of these, Hsp70 (70 kDa) and Hsp90 (90 kDa) are the most used classes for stress studies.

**Figure 9:**
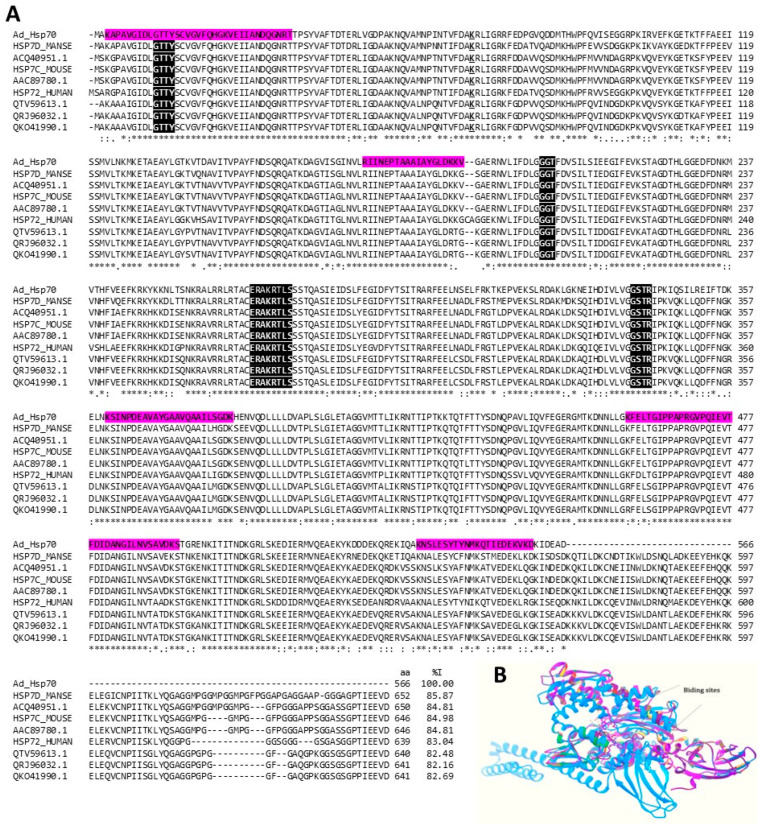
**Hsp 70 kDa**. (**A**) Multiple alignments of the amino acid sequence of putative *A. dowii* Hsp 70 (Ad_Hsp70 (Transcriptome: c27026_g1_i1)) with other proteins of the same family. In the alignment, the sequences Hsp70 from insects (UniProtKB: HSP7D_MANSE), from humans (UniProtKB: HSP72_HUMAN), from mice (UniProtKB: HSP7C_MOUSE), and several patented Hsp70s for different applications (GenBank: ACQ4089780.1, A QTV59613.1, QRJ96032.1, QKO41990.1). The region covered by the tryptic peptides obtained from the proteomes of *A. dowii* and *L. neglecta* is highlighted by a magenta background. The ATP binding site is underlined and the regions involved in the binding of nucleotide phosphates are highlighted with white letters on a black background. (**B**) Structure models of heat shock proteins: Ad_Hsp70 is green, HSP7D_MANSE is medium blue, ACQ40951.1 is blue, HSP7C_MOUSE is forest green, AAC89780.1 is gray, HSP72_HUMAN is pink, QTV59613.1 is yellow, QRJ96032.1 is red, and QKO41990.1 is purple. The comparison of the Cα chains presented RMSD values that oscillate between 0.007 and 0.77.

Hsp70 has been used as an early stress response biomarker for several marine organisms [74], including some cnidarian species such as *Nematostella vectensis* and *Aurelia coerulea* [40,49]. HSPs with a molecular weight of 70 and 90 kDa have also been shown to function as tumor-derived immunogenic peptide carrier proteins that induce a T cell-mediated immune response against cancer [75]. Furthermore, several studies have reported a mammalian neuroprotective effect of Hsp70, particularly after induction or administration [76]. It has also been reported that Hsp60 and Hsp70 can prevent the oligomerization of amyloidogenic proteins [77]. In the proteomes of *A. dowii* and *L. neglecta*, sequences have been identified that code for probable chaperones Hsp60, Hsp90, and Hsp70. The amino acid sequence of the Hsp70 precursor was identified in the *A. dowii* transcriptome and showed 82.1 and 85.7% identity with several Hsp70s that are part of inventions with potential medical and biotechnological uses (Figure 9A).

The secretion of over-expressed proteins in yeast is increased by the design of gene constructs that express Hsp70 (AAC89780.1) [78], whereas Hsp70 (QTV59613.1) stimulates cholesterol efflux from macrophage foam cells and thus has potential for use in controlling cholesterol homeostasis [79]. Furthermore, Hsp70 (ACQ40951.1) can stimulate the proliferation and cytolytic activity of Natural Killer (NK) cells against tumor cells [80]. In another application, an immunogenic peptide of between eight and fifteen amino acid residues was generated from Hsp70 (QKO41990.1); this peptide induces cytotoxic T lymphocytes against tumor cells via antigen presentation [81]. Finally, an invention that involves a mutant of Hsp70 (QRJ96032.1) that lacks the dendritic cell-binding site is useful for the treatment of vitiligo [82].

Our structural alignment (Figure 9B) shows that the studied HSPs maintain the characteristic structure of HSP70, made up of a nucleotide-binding domain and a protein-binding domain [83]. Except for the HSP7D_MANSE protein that presents an insertion between residues 221 and 421, the other HSPs do not show relevant alterations.

#### 2.3.5. Serine Protease Inhibitors (Serpins)

**Figure 10:**
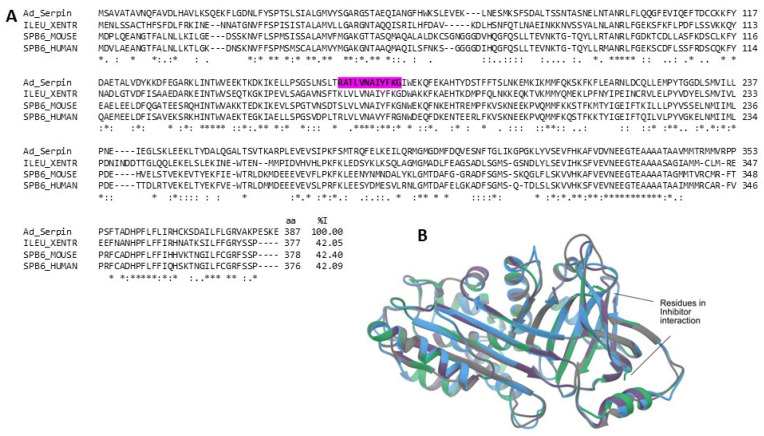
Serpin alignment. (**A**) Multiple alignment of the amino acid sequence of the putative serine protease inhibitor (Ad_Serpin (Transcriptome: c26903_g1_i1)) with the serpins of humans (UniProtKB: SPB6_HUMAN) and mice (UniProtKB: SPB6_MOUSE), and the elastase inhibitor identified in frogs (UniProtKB: ILEU_XENTR). The region covered by the tryptic peptides obtained from the *A. dowii* and *L. neglecta* proteomes is highlighted by a magenta background. (**B**) Structural models of protease inhibitors: Ad_Serpin is blue, ILEU_XENTR is gray, SPB6_MOUSE is green, and SPB6_HUMAN is purple. The comparison of the Cα chains presented RMSD values that oscillate between 0.361 and 0.863.

Serine proteases play a fundamental role in blood coagulation, fibrinolysis, complement activation, and inflammation [84]. The excessive activity of these enzymes can trigger pathological states, so it is necessary to regulate their activity. Serpins (serine protease inhibitors) are proteins of ∼350–400 amino acid residues, responsible for regulating serine proteases’ function through stoichiometric binding to the active site, inactivating their action. Some serpins are secreted and act extracellularly, with some exceptions, such as the Ov-serpin subfamily, which is structurally related to ovalbumin and lacks a signal peptide for its secretion; therefore, members of this subfamily can also act extracellularly. Intracellular serpins have shown the ability to inhibit proteolytic activity during inflammation to act as modulators of the inflammatory response, which makes them candidates for the development of pharmacological formulations for treating inflammatory diseases such as rheumatoid arthritis [85]. We identified the sequence of a likely member of the Ov-serpin subfamily, which we named Ad_Serpin. This probable protein was identified in the CVE of both species and the *A. dowii* tentacle transcriptome. The Ad_Serpin sequence presents 42.05 and 42.40% similarity with human, frog, and mouse serpins (Figure 10A). Figure 10B shows that protease inhibitors maintain the structure of serine protease inhibitors. In addition to general folding, residues that allow for inhibitor interactions, such as Ser195 andCys347, are conserved [86].

## 3. Discussion

The development of new molecules from natural products has grown exponentially in recent decades. Searching for new molecular alternatives to solve health problems and facilitate industrial processes has become attractive. The ocean is an excellent source of natural products due to its biological diversity. Marine bioprospecting has led the way in the exploration of all kinds of molecules with potential applications in various industries and biotechnology, which has allowed us to go beyond the search for “classic” or “more attractive” molecules to molecules that are part of the metabolism and general physiology of organisms of all kingdoms. In this direction, there is a relatively considerable increase in the study of molecules that show a notable difference in their production when organisms inhabit environments whose physical, chemical, and biological parameters can change drastically in one day, which generates immediate adjustments in their metabolism, implying that the production of enzymes and other molecules is affected. During the evolution of these organisms, molecules, such as enzymes that allow for the rapid adaptation of organisms, have modified their biochemical properties to respond to environmental changes. These new biochemical characteristics are attractive for use as molecular tools in biotechnology.

Marine invertebrates are the top group of natural compounds, with attractive applications. Sea anemones are one of the least explored invertebrates from the perspective of marine bioprospecting. These organisms are sessile and have limitations to their ability to move in response to imminent environmental changes that challenge their physiology. However, they can colonize all marine habitats and belong to one of the oldest phyla, with an estimated age of approximately 750 million years [87], suggesting that they have efficiently adapted their molecular machinery to survive environmental changes. The temperatures and UV radiation to which anemones can be exposed and the symbiotic interactions they establish with photosynthetic microalgae generate large amounts of ROS that sea anemones can effectively manage. Sea anemones are still considered unusual species for the bioprospecting of non-toxic molecules to develop biopharmaceutical products. However, proteomic and transcriptomic studies focusing on their venom [31,32,38,87,88], as well as their symbiotic interactions, have revealed the presence of proteins with potential activities that could be evaluated and taken to the second stage to estimate their biotechnological potential. For this reason, we led this marine bioprospecting study in two species of anemones from different geographical areas through proteomic analysis. It was determined that the anemone *A. dowii* that lives in an area with more significant environmental changes, during its physiological adjustments, produces more proteins that respond to stress, while *L. neglecta* produces more antioxidant proteins, possibly due to changes in pH and temperature in its environment. This can be used to determine that metabolic adjustments are evident in environments that are as changing as the interstitial zone. The proteins identified in both proteomes included hydrolase-type enzymes, oxidoreductases, heat shock proteins, adhesion proteins, structural proteins, and protease inhibitors. The presence of these enzymes in *A. dowii* might be explained by the physical–chemical stressors it encounters. However, they contradict the hypothesis that the elevated expression of antioxidant proteins in *L. neglecta* is solely due to radiation and temperature, suggesting that it could be associated with photosynthetic algae living in symbiosis with *L. neglecta*. Such interactions have been shown to promote the production of novel biomolecules of pharmacological interest in holobionts [42], indicating that the environment plays a significant role in shaping the composition of biomolecules in sea anemones.

Several proteins from other organisms have been described in depth, with the possibility of their application in medicine or biotechnology. However, it is essential to carry out experimental studies of the polypeptides identified here to determine the degree of potential for their application.

## 4. Materials and Methods

### 4.1. Sea Anemone Collection

*A. dowii* was collected during low tide in the intertidal zone of the Pacific Ocean coast in Ensenada, Baja California, Mexico, and *L. neglecta* was collected at a 10 m depth in the front reef zone of Puerto Morelos, Quintana Roo, in the Mexican Caribbean. Sea samples were collected and transferred to the laboratory in seawater and then frozen at −80 °C until use.

### 4.2. Protein Extraction

The *A. dowii* crude venom extract was prepared from three organisms, as described in [89], and *L. neglecta* crude venom extract included five complete individuals that were thawed and deposited in 120 mL of 50 mM sodium phosphate at pH 7.4 with protease inhibitors and mixed with constant stirring at 4 °C for 12 h to stimulate the discharge of the nematocysts. Then, the crude venom extract was subjected to three rounds of freezing and thawing to enhance nematocyst discharge. The suspension was centrifuged at 25,400× *g* for 40 min to remove cell debris. The crude venom extract was filtered through sterile 0.22 µm membranes, and the protein was quantified by the Bradford assay [90]. The analysis of the crude venom extracts electrophoretic profiles used SDS–PAGE in 15% acrylamide [91].

### 4.3. Proteomic Samples

Protein samples from the crude venom extracts of *A. dowii* and *L. neglecta* were prepared in triplicate for analysis by Shotgun proteomics. All replicates contained 40 µg of total protein in 100 µL TE buffer (10 mM Tris–HCl pH 7.6, 1 mM EDTA pH 8) and 100 µL of 0.3% sodium deoxycholate [*w*/*v*], totaling 300 μL with sterile tetra distilled water. The samples were precipitated with 72% (*w*/*v*) trichloroacetic acid (TCA) for 3 h on ice and subsequently centrifuged at 8436× *g* for 20 min at 4 °C. The pellets were recovered and subjected to additional precipitation with 90% (*v*/*v*) acetone overnight at −30 °C. The samples in acetone were centrifuged at 8436× *g* for 20 min at 4 °C, the pellet was dried for 20 min in a Savant integrated SpeedVac system (Thermo Fisher Scientific, Waltham, MA, USA), and the supernatant was discarded. The samples were sent to the proteomics unit of the Institute de Recherches Cliniques (Montreal, QC, Canada) for processing.

### 4.4. Protein Digestion

The protein samples were resuspended with 10 μL of 6 M urea and reduced with 45 mM DTT for 30 min at 37 °C. Subsequently, the samples were alkylated with 55 mM iodoacetamide for 20 min at 24 °C in the dark. Each of the samples was diluted with 18.2 Mohm purified water (Merck Millipore, Darmstadt, Germany) H_2_O until the urea concentration was 2 M. Then, the samples were digested with 10 μL trypsin solution (5 ng/μL Promega sequencing-grade trypsin, 50 mM ammonium bicarbonate); after 18 h, the reaction was stopped with 5% formic acid. The samples were centrifuged in a vacuum system until drying, then stored at −20 °C until use. Tryptic peptides were resolubilized by shaking in 10 μL of a solution of 0.2% formic acid for 15 min and desalting using C18 ZipTips pipette tips (Millipore, Billerica, MA, USA). The eluted samples were vacuum-dried and resolubilized by stirring for 15 min in 10 μL of a 2% acetonitrile/1% formic acid solution.

#### LC–MS/MS

Trypsinized samples were analyzed by LC–MS/MS using an Easy-nLC II liquid chromatography system (Proxeon Biosystems, Odense, Denmark) coupled with an Orbitrap Velos LTQ mass spectrometer (ThermoFisher Scientific, Bremen, Germany) using a nano-electrospray (Proxeion) ion source. Chromatography was performed using a 15 cm long, 75 μm internal diameter Self-Pack PicoFrit column, fused to a silica capillary (New Objective, Woburn, MA, USA) and packed with C18 Jupiter 5 μm 300 materials and ÅRP (Phenomenex, Torrance, CA, USA). The buffers used for chromatography were 18.2 Mohm purified water (Merck Millipore) H_2_O at 0.2% formic acid (buffer A) and 100% acetonitrile/0.2% formic acid (buffer B). Samples (5 μL) were loaded onto the column at a 600 nL/min flow rate and eluted with a 2-step linear gradient at a 250 nL/min flow rate. Buffer B increased from 2 to 35% in 80 min and then from 35 to 80% in 12 min. LC–MS/MS data acquisition was achieved using a parallel acquisition mode, where the full scan MS data were acquired in the orbitrap (R = 60,000), and the top 15 precursors were isolated and fragmented in the linear ion trap. MS spectra were acquired over *m*/*z* 360 to 1700 and used an AGC of 1,000,000. MS/MS spectra were subject to the 1/3 (of the precursor *m*/*z*) rule for ion traps and used an AGC of 1,000,000 loadings. Data-dependent scan events used a maximum ion fill time of 100 ms and 1 Microscan. Target ions selected for MS/MS were excluded for 31 s after 2 counts. The nanospray and S-lens voltages were set to 1.3–1.8 kV and 50 V, respectively. The capillary temperature was adjusted to 250 °C. MS/MS conditions were normalized; the collision energy occurred at 35 V; activation q was 0.25; activation time was 10 ms.

### 4.5. Identification and Quantification of Proteins

Tandem mass spectra were extracted using Mascot Daemon v2.1 (Matrix Science, London, UK). Deconvolution and deisotope of the state of charge were not performed. The protein search was performed against the Refseq_Cnidaria database. The precursor ion mass width was set at ±10 ppm and the maximum fragment mass error was set to 0.6 Da. Trypsin was assumed to be the digesting enzyme, and up to 1 cleavage was allowed to be skipped. Cysteine carbamidomethylation was chosen as a constant modification, and methionine oxidation was determined as a variable modification. MS/MS-based polypeptide identifications were validated with Scaffold software v4.11.1 (Proteome Software Inc., Portland, OR, USA). Scaffold identification parameters were set to at least two unique peptides per protein, with a minimum probability of 99% for proteins [92] and 95% for corresponding peptides [93]. Proteins containing similar peptides that could not be differentiated by MS/MS analysis were only pooled to satisfy parsimony principles.

To compare the proteomic information between both species of sea anemones, quantitative analyses of the peptides without labeling were carried out using the spectral counting method and analyzed using the exponentially modified protein abundance index (emPAI) using the Scaffold software (see Supplementary Material S1). Protein expression was considered significantly different only if the protein ratios differed two-fold, and the volcano plot shows the changes in protein expression. Student’s *t*-test was applied to each set of samples with three different biological replicates, and a threshold level of 0.05 was considered to select proteins (Supplementary Material S1). All data were corrected by applying the Benjamini-Hochberg correction. The distribution of the spectra and total unique peptides, as well as the number of proteins identified exclusively in each species and those identified in both, were obtained, and are presented graphically in Venn diagrams. The proteomic indices of both species, such as the emPAI, amino acid sequence coverage, and molecular weight distribution of the identified proteins, were extracted from the Scaffold software and represented in bar graphs. All proteomic analysis graphs were performed with R (www.r-project.org (accessed on 16 July 2020)) and Rstudio (www.rstudio.com (accessed on 17 July 2020)).

### 4.6. Bioinformatic Analysis

The total proteins identified in each extract were annotated if they met the scaffold parameters mentioned above (Section 4.5) and were identified as having two or more unique peptides in at least two of three biological replicates. For the functional annotation of previously identified proteins in the Refseq_Cnidaria database, each protein was manually curated using BLASTP (https://blast.ncbi.nlm.nih.gov/Blast.cgi, accessed on 4 September 2021) and the UniProtKB data database (https://www.uniprot.org/; accessed on 4 September 2021). In addition, a homemade database generated from the information obtained from the *A. dowii* tentacle transcriptome, previously reported by our group [31,44], was used; this allowed us to identify the complete sequence and improve the annotation process using the UniProtKB database. The functional assignment, cellular location, and biological processes related to the total proteins identified in *A. dowii*, *L.neglecta*, or both samples were obtained from the QuickGO Gene Ontology database (https://www.ebi.ac.uk/QuickGO/; GO version: 4 September 2021) and were presented with Excel 15.28. For some proteins identified in the crude venom extract of *A. dowii*, the restriction to a single peptide present in at least two biological replicates was modified, maintaining the percentage probability of identification of peptides and proteins established for Scaffold, if the protein was also identified in the transcriptome of *A. dowii* [31,44].

Protein–protein interactions between all proteins identified in *A. dowii* and *L. neglecta* proteomes were analyzed for the proteins identified in each crude venom extract using the String v11.5 software (https://string-db.org/, accessed on 4 September 2021). The *N. vectensis* genome database was used as a template, and these data contributed to the identification of each protein, along with its metabolic pathway. The interactions were generated according to the functional and physical relationship between the proteins. The clustering of interactions in *A. dowii* and *L. neglecta* was associated using the Markov Clustering Algorithm (MCL) clustering method using an inflation parameter of 2 [94]. The network edges were set confidentially (the thick line represents the strength of the data support), and the sources of interaction were text mining, databases, experiments, co-expression, neighborhood, gene fusion, and co-occurrence.

Proteins with differential downward or upward expression, identified in both extracts (Section 4.5), were also analyzed and grouped according to their expression and molecular function. The protein–protein interactions obtained with the String v11.5 software identified the metabolic pathways or processes in which the proteins participate that alter their expression; the minimum interaction score required for all analyses in String v11.5 was set at 0.7 (high confidence) [95]. Multiple alignments of some selected sequences from the sea anemone proteomes were analyzed against functionally and structurally characterized orthologs. Alignments were performed with Clustal Omega (1.2.4) (https://www.ebi.ac.uk/Tools/msa/clustalo/, accessed on 4 September 2021). The number of amino acids (aa) and the identity percentages (I%) were calculated considering the total sequence. In each alignment, the characters (*), (:), and (.) indicate positions with fully conserved residues, positions with conservative substitutions, and positions with less conservative substitutions, respectively. The same proteins in the alignment were modeled with SWISS-MODEL [96]. Each model was minimized with YASARA [32]. Finally, the structures were refined using 3D refinement [97] and analyzed using UCSF Chimera [98].

## Data Availability

The data generated is Supplementary Material.

## Supplementary Materials

The following supporting information can be downloaded at https://www.mdpi.com/article/10.3390/md22010012/s1, S1, Putative components identified in the crude venom extract in the proteome from *Anthopleura dowii* Verrill, 1869, and *Lebrunia neglecta* Duchassaing & Michelotti, 1860. S2–S4, Interactomes. S5, Differentially expressed proteins. S6, Interactome plot.
