# Supplementary material for: Marine Bioprospecting: Enzymes and Stress Proteins from the Sea Anemones Anthopleura dowii and Lebrunia neglecta
Source: Mar Drugs. 2023 Dec 23;22(1):12. doi: 10.3390/md22010012 (PMC10821040; doi:10.3390/md22010012)
Supplement: Supplementary file 1 [file marinedrugs-22-00012-s001.zip › Supplementary material S6.pdf]

Supplmentary material S6. Protein-protein interaction network

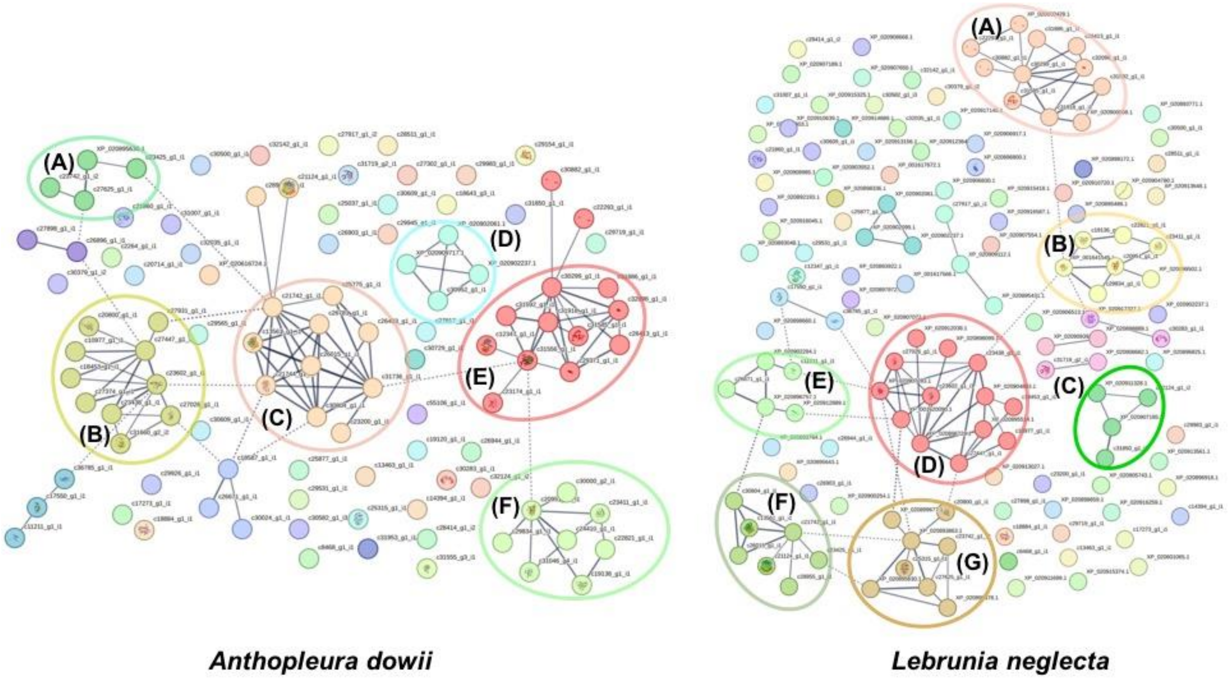

S6. Protein-protein interaction network. The interactome was built with String v11.5 software of proteins found in crude extracts of *A. dowii* and *L. neglecta*. The circles indicate the interaction nodes. The number of nodes obtained in the interaction network for the *A. dowii* proteome was 103, while for *L. neglecta* it was 142 nodes. In both interactomes, a PPI enrichment p-value < $1.0e^{-16}$ was obtained.
